# Anthropometric Measures and Risk of Rheumatoid Arthritis in the French E3N Cohort Study

**DOI:** 10.3390/nu14050934

**Published:** 2022-02-22

**Authors:** Carine Salliot, Yann Nguyen, Xavier Mariette, Marie-Christine Boutron-Ruault, Raphaèle Seror

**Affiliations:** 1 Centre for Research in Epidemiology and Population Health (CESP), INSERM U1018, Paris-Saclay University, 94800 Villejuif, France; carine.salliot@chr-orleans.fr (C.S.); yann.nguyen2@aphp.fr (Y.N.); 2 Rheumatology Department, Centre Hospitalier Régional d’Orleans, 45067 Orléans, France; 3 Centre of Immunology of Viral Infections and Auto-Immune Diseases (IMVA), INSERM U1184, Paris-Saclay University, 94270 Le Kremlin Bicêtre, France; xavier.mariette@aphp.fr (X.M.); raphaele.seror@aphp.fr (R.S.); 4 Department of Internal Medicine, AP-HP-Hôpital Beaujon, Paris University, 92110 Clichy, France; 5 Rheumatology Department, AP-HP-Hôpital Bicêtre, Paris-Saclay University, 94270 Le Kremlin Bicêtre, France

**Keywords:** rheumatoid arthritis, prospective cohort, risk factor, anthropometric measures, body shape trajectories

## Abstract

We aimed to assess the relationships between anthropometric measures and risk of rheumatoid arthritis (RA). The E3N cohort included 98,995 women (aged 40–65 years at the recruitment) who completed mailed questionnaires on reproductive factors, lifestyle, and health-related information, including anthropometric measures, every 2–3 years. Cox proportional hazards regression models with age as the time scale and adjusted on known RA risk factors were used to estimate hazard ratios (HRs) and 95% confidence intervals for the risk of incident RA in the overall population (*n* = 78,452) and after stratification on smoking exposure. Incident RA diagnosis was validated in 698 women. Abdominal obesity (waist circumference >88 cm) was associated with RA (HR = 1.2 (1.0–1.5)), independent of BMI; whereas obesity, defined as BMI ≥ 30 kg/m^2^, was marginally associated with RA (HR = 1.26 (0.9–1.5), *ptrend* = 0.0559). Taking lean body shape (BS) as reference, medium BS at puberty (HR = 1.3 (1.0–1.7)) and medium-large BS at perimenopausal period (HR = 1.5 (1.1–1.9)) were associated with the risk of RA among never-smoker women, independent of BMI. Regarding BS trajectory, taking constantly lean BS as reference, constantly large BS from puberty to perimenopause was associated with RA among non-smokers (HR = 2.10 (1.2–3.6)), independent of BMI.

## 1. Introduction

Rheumatoid arthritis (RA) is the most common inflammatory rheumatic disease. Its prevalence is estimated of 0.5%. Both environmental and genetic factors are involved in its pathophysiology. They interact in the pathogenesis by triggering autoimmunity [1]. Some alleles of HLA-DRB1 (so called shared epitope) are associated with the development of a subset of RA characterized by presence of ACPA (auto-antibodies directed against the citrullinated peptide) [2]. These autoantibodies could be detected years before RA onset (so called preclinical stage). This suggests that the autoimmune process starts several years before the first symptoms of the disease. Active smoking has been reproducibly and strongly associated with an increased risk of ACPA-positive RA, especially in individuals carrying the shared epitope [1,2].

Fat tissue is well-known to secrete some pro-inflammatory cytokines. An excess of fat tissue is accompanied by a chronic inflammation state and may increase the risk of chronic inflammatory diseases [3]. Indeed, some case–control and cohort studies suggested that overweight and/or obesity could be associated with risk of RA, mainly in women and seronegative RA [4,5,6,7,8]. Crowson et al. estimated that worldwide increasing prevalence of obesity may explain 52% of the last decades’ increase in RA incidence [9]. Moreover, some data suggested that overweight/obesity may be associated with the occurrence of RA, independent of smoking [6]. In all these studies, body mass index (BMI) has been the preferred measure of body fat, although it does not reflect fat distribution and visceral fat. Other anthropometric measurements such as waist and hip circumferences and bioimpedance-derived body fat percentage have been rarely assessed as risk factors of RA [10]. Another measure that could be of interest since it provides both information on body fat and its distribution is body shapes or silhouette. In addition, changes in these anthropometric measures throughout life have never been assessed in relation to the risk of RA but could be of interest to better understand the associations between fat tissue distribution changes and the risk of subsequent RA. For these reasons, it seems pertinent to investigate the impact of anthropometric measure variations throughout life on the risk of RA by assessing the impact of body shape trajectories from childhood to perimenopause. Some of the body shape trajectories have been shown to be associated with an increased risk of type 2 diabetes, depression, and breast cancer in the E3N French women cohort [11,12,13].

Our objective was to assess the association between anthropometric measures, including body shapes and their lifetime trajectories, and the risk of incident RA in the E3N prospective cohort of French women.

## 2. Materials and Methods

### 2.1. The E3N Study 

E3N (“Etude Epidémiologique auprès des femmes de la Mutuelle générale de l’Education Nationale”) is a French prospective cohort study including 98,995 healthy French women. They were born between 1925 and 1950 and affiliated to a national health insurance plan for workers of the French education system and their families [14]. Since recruitment in 1990, all participants completed biannual mailed questionnaires (to date, 12 questionnaires were completed) to update their lifestyle characteristics, health-related information, and newly diagnosed diseases. Since 2004, a linkage with the drug reimbursement claim database from medical insurance records (Mutuelle Générale de l’Éducation Nationale (MGEN)) has been available. The average response rate per follow-up questionnaire is 83%, and the proportion of women lost to follow-up since recruitment is very low (<3%). All participants gave their written informed consent at recruitment. Approvals were obtained from the French National Commission for Data Protection and Individual Freedom (327346-V14) and the French Advisory Committee on Information Processing in Material Research in the Field of Health (13.794).

### 2.2. Validation of RA Cases and Study Population

The validation of RA cases among E3N women was previously described [15]. Briefly, in 2017, a specific questionnaire for inflammatory rheumatic disease (IRD) validation was sent to all women who self-reported having RA (N = 2692) in three of the follow-up questionnaires (2008, 2011, and 2014) or in any questionnaire if they reported a hospital admission for RA. Women were considered RA cases if they confirmed having RA in the specific questionnaire and fulfilled any of the following criteria: (1) RA confirmed by a physician; (2) they self-confirmed taking or having taken any RA-specific disease-modifying anti-rheumatic drugs (DMARDs) or biological treatments; (3) they self-confirmed having RA with either rheumatoid factor (RF) or ACPA; or (4) RA met at least 4 of the 7 criteria of the 1987 American College of Rheumatology criteria classification. In addition, women who did not answer the specific IRD questionnaire were considered to have validated RA if they self-reported having RA and had reimbursements of any RA-specific medication (DMARDs or biologics) using the MGEN medication reimbursements database [16]. For the present study, we excluded women who had other IRD or prevalent RA (i.e., already present at baseline) or without date of RA diagnosis and who had never menstruated. 

### 2.3. Assessment of Anthropometric Measurements

In each follow-up questionnaire, participants were asked to report their weight. Height was collected in 1990 and 1995 and was regularly updated since 2000. BMI was calculated at baseline and for each follow-up questionnaire until Q11 as weight (kg)/[height (m)]^2^ and was expressed in kg/m^2^. According to WHO (World Health Organization), BMI was categorized as the following: <18.5 kg/m^2^ (underweight), 18.5–25 (normal), 25–30 (overweight), and ≥30 kg/m^2^ (obesity). Self-reported hip and waist circumferences, measured according to precise instructions, were collected in the 1995 (Q4), 2002 (Q7), 2005 (Q8), 2011 (Q10), and 2014 (Q11) questionnaires. Hip circumference was defined as the largest circumference below the umbilicus, while waist circumference was defined as the smallest circumference between the base of the ribs and the largest point of the iliac crest [17]. These measures were categorized in quartiles. Waist-to-hip ratio (WHR) was computed as waist circumference/hip circumference. Abdominal obesity was assessed according to two different WHO definitions available for women: a waist circumference >88 cm or a WHR >0.85 in women [18]. 

**Figure 1:**
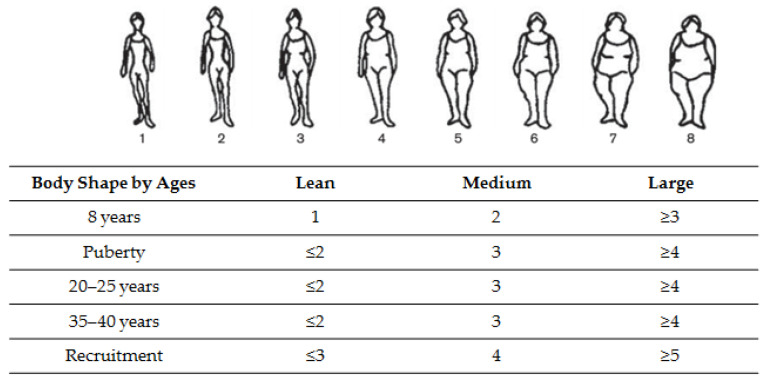
Classification of body shape at different ages, using Sørensen’s pictograms [19].

Age-related body shapes and body shape trajectories throughout life. In the first questionnaire and using the eight Sørensen body shapes, women reported which drawing best described their body shape at around the age of 8 years, at puberty, at ages 20–25, 35–40, and at recruitment (i.e., at perimenopausal period) (Figure 1) [19]. These eight body shapes represented extreme thinness to obesity and were previously validated in the E3N cohort [11,12,13,17,20]. For each of these age-related body shapes, we created three categories (lean, medium, and large) using a different classification according to the period of life (Table of the Figure 1). 

### 2.4. Covariates 

Educational level (<high school, up to 2 years of university, and ≥3 years of university), passive smoking (during childhood and/or adulthood, ever/never), physical activity (in metabolic equivalents of task, hours/week), age at menarche, and number of full-term pregnancies were collected at the first or second questionnaire. Active smoking status (past, current, and never-smoker), and age at menopause were collected at baseline and regularly updated. The relationship between all of these covariates and the risk of RA was previously described [16,21]. 

### 2.5. Statistical Analyses

Baseline women’s characteristics are presented as mean (standard deviation (SD)) and in quartiles for continuous variables and n (%) for categorical variables. Woman-years for each category of exposure were estimated from the recruitment to the occurrence of RA, death, or the last completed questionnaire (up to the 2014 questionnaire), whichever occurred first.

Body shape trajectories were constructed using Nagin’s approach to group-based trajectory modeling [22,23]. This method is an application of finite mixture modeling and enabled us to define clusters of women with a similar evolution of body shape over time. Trajectories were evaluated using the censored normal model of the SAS Proc Traj. The optimal number of groups and shapes of trajectories was selected to best fit the data, as evaluated by a change in the Bayesian information criterion and the percentage of individuals included in each category. Six body shape trajectories were finally estimated with a cubic function of age, as previously described in the E3N cohort [11,12,13].

Hazard ratios (HRs) and 95% confidence intervals (95% CIs) were estimated using Cox proportional hazards regression models, with age as the time scale and stratification according to year of birth (“before 1930”, “1930–1935”, “1935–1940”, “1940–1945”, and “after 1945”). Cox regression models were first age-adjusted (model 1), then adjusted for known risk factors of RA, i.e., active smoking, passive smoking, educational level, and BMI if applicable (model 2). Multi-adjusted models were additionally adjusted for ages at menarche, at menopause, number of full-term pregnancies [21], and baseline physical activity and included anthropometric measures significantly associated with RA in models 1 and/or 2. Smoking, waist and hip circumferences, and BMI were time-dependent variables in the Cox regression models. When relevant, we performed tests for linear trend by using an ordinal score for each exposure. In addition, because smoking exposure is a major risk factor for seropositive RA, we stratified our analyses according to this exposure (passive smoking during childhood and/or active smoking during adulthood; ever vs. never). 

Women with missing data for each anthropometric factor were excluded from Cox regression models. For potential confounders, missing values, occurring in <5% of observations, were imputed to the median (continuous variables) or to the modal category (categorical variables), which has proven to be satisfactory (low risk of bias) in the E3N cohort [24]. Otherwise, a missing category was created. 

All statistical analyses were performed using SAS v9.4 (SAS Institute Inc., Cary, NC, USA). All statistical tests and corresponding *p*-values were 2-sided. A *p*-value < 0.05 was considered statistically significant. 

## 3. Results

### 3.1. Study Population 

**Figure 2:**
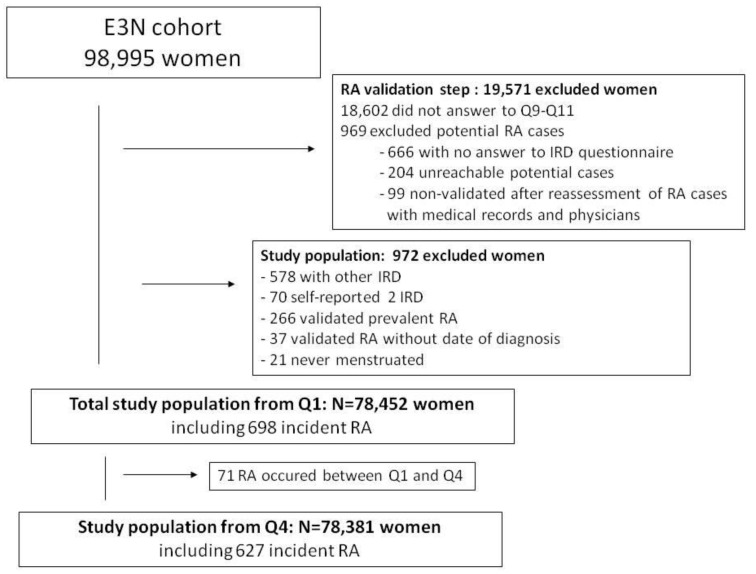
Study population in the E3N cohort. IRD: inflammatory rheumatic disease, RA: rheumatoid arthritis.

A total of 78,452 women, having a mean age of 49 years at recruitment, were included in the overall population, for a total follow-up of 1,865,213 women-years. A total of 698 incident cases of RA occurred after recruitment (Figure 2). Table S1 shows the main characteristics of the population and RA cases. 

### 3.2. RA Characteristics 

The mean age at RA diagnosis was 63.7 (±9.0) years. ACPA/RF status was available for only 229 (32.8%) cases: 209 (91.2%) were seropositive and 20 (8.7%) seronegative. The mean follow-up from RA onset to the last questionnaire available was 10.1 ± 6.6 years. Treatments received for incident RA were available in 505 women (72.3%): 429 RA women received methotrexate (85%), 88 had a TNFα inhibitor (17.4%), and 39 (7.7%) other biologics (rituximab, abatacept, tocilizumab).

### 3.3. Associations between Anthropometric Measures and Risk of RA 

#### 3.3.1. Obesity and Abdominal Obesity

Obesity (BMI ≥ 30 kg/m^2^) was significantly associated with the risk of RA (HR = 1.33 (1.0–1.8), *ptrend* = 0.0263) in the age-adjusted model, taking normal BMI (18.5–25 kg/m^2^) as reference (Table S2). This association remained after adjustment for smoking exposure (HR = 1.30 (95% CI 1.0–1.7), *ptrend* = 0.0445, Table S2) but was no longer significant in multi-adjusted models or in the overall population (HR = 1.26 (0.9–1.5), *ptrend* = 0.0559) or in analyses stratified on smoking status. However, the magnitude of HR in ever-smokers remained high (HR = 1.35 (CI 0.9–1.9)) compared with never-smokers (HR = 1.15 (0.7–1.8)) (Table 1). 

Abdominal obesity (waist circumference >88 cm) was significantly associated with RA in all models (HR = 1.25 (1.0–1.5), *p*-value = 0.0338 in multi-adjusted model) (Table S2 and Table 1), independent of smoking status and BMI. The association was no longer statistically significant in stratified analyses due probably to lack of power, since HRs remained in the same range as in the overall population in ever-smokers (HR = 1.32 (0.9–1.8)), but was lower in never-smokers (HR = 1.12 (0.8–1.6)). Abdominal obesity according to the waist-to-hip circumference ratio definition was not associated with RA (Table S2).

#### 3.3.2. Body Shapes and Their Trajectories

BS at 8 years, 20–25 years, and 35–40 years was not associated with the risk of RA (Table S2), whereas BS at puberty was associated with the risk of RA, independent of BMI and perimenopausal BS. Indeed, medium BS at puberty was significantly associated with RA in all models (Table S2 and Table 2), taking lean BS as reference (HR = 1.22 (1.0–1.5) in Table 2 multi-adjusted model). In stratified analyses, this association was statistically significant only among never-smokers (HR = 1.30 (1.0–1.7)), but not among ever-smokers (HR = 1.17 (0.9–1.5)) (Table 2). In age-adjusted model 1, there was also a significant positive relationship between medium (HR = 1.20 (1.0–1.4)), and large (HR = 1.32 (1.1–1.6)) perimenopausal BS and RA risk (*ptrend* = 0.0055), taking lean BS as reference (Table S2). 

The association between large BS at perimenopause was independent of known risk factors for RA, such as smoking (HR = 1.30 (1.0–1.6), *ptrend* = 0.0103 after adjustment on smoking status), but disappeared after adjustment for pre-diagnosis BMI (HR = 1.26 (0.9–1.6), *ptrend* = 0.0637) (model 2 in Table S2). 

In the multi-adjusted model including both BS at puberty and at perimenopause, medium BS at perimenopause was significantly associated with RA only among never-smokers (HR =1.46 (1.1–1.9), lean BS as reference). Large BS was no longer associated with RA among never-smokers, probably due to lack of power in this category since HR remained in the same range and *ptrend* was statically significant (HR = 1.45 (0.9–2.2), *ptrend* = 0.0375) (Table 2).

**Figure 3:**
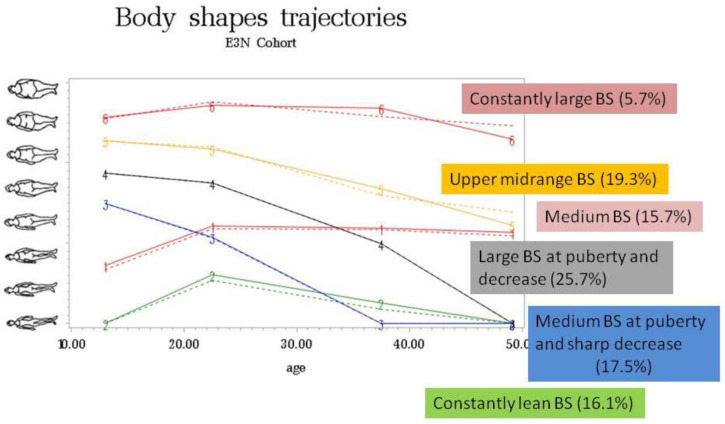
Trajectories of self-reported body shapes (BS) from puberty to baseline.

To build BS trajectories, the study population included 77,552 women (687 incident RA), after the exclusion of women with missing information on BS at all time points (*n* = 900). Six body shape trajectories from puberty to perimenopausal period (i.e., baseline) were built and defined as: “constantly lean BS” (16.1%), “medium BS at puberty and sharp decrease in BS after puberty” (17.5%), “large BS at puberty and decrease in BS after puberty” (25.7%), “constantly medium BS” (15.7%), “upper midrange BS” (19.3%), and “constantly large BS” (5.7%) (Table S1 and Figure 3).

Taking “constantly lean” BS trajectory from puberty to perimenopausal period as reference, “constantly large” BS trajectory was significantly associated with the risk of incident RA in the age-adjusted model (HR = 1.42 (95% CI 1.0–2.0)) (Table S2). This association was independent of known risk factors for RA, such as smoking (HR = 1.40 (1.0–1.9)), but disappeared after adjustment for pre-diagnosis BMI (HR = 1.31 (0.9–1.9), model 2 in Table S2) and in multi-adjusted model 3 (HR = 1.29 (0.9–1.9), Table 3). After stratification on smoking exposure, “constantly large” BS trajectory was significantly associated with RA, independent of BMI, but only among never-smokers (HR = 2.10 (1.2–3.6), *ptrend* = 0.0248), taking “constantly lean” BS as reference (Table 3).

## 4. Discussion

In this large prospective cohort of women, and for the first time, we found that anthropometric measures that reflect fat distribution variations throughout life were associated with an increased risk of incident RA, especially among participants never exposed to smoking and independent of BMI: medium BS at puberty, medium-large BS at perimenopausal period, and constantly large BS trajectory from puberty to perimenopausal period. Moreover, adulthood abdominal obesity was also associated with RA, independent of smoking exposure, whereas obesity (BMI ≥30 kg/m^2^) was not significantly associated with RA after multi-adjustments. 

In numerous studies, overweight or obesity has been found to be independently associated with an increased risk of RA, both in case–control [4,5,6] and cohort studies [7,10,25], except in the Iowa Women Health Study (no association) [8]. The positive association between obesity and RA was restricted to women or to seronegative RA in the majority of studies [5,10,26]. Thus, our results, restricted to women never exposed to smoking, are in line with these data, as smoking is highly associated with ACPA [1,2]. However, in the Nurses’ Health Study (NHS), elevated BMI and abdominal obesity were positively associated with seropositive RA, with a possible synergistic effect between elevated BMI and positive ACPA status on the risk of RA [7,25,27]. Moreover, in a cohort of pre-RA, the risk increased to 60% if smoking history and overweight were combined (2% in never-smokers with normal weight) [28]. 

A high BMI at age 18 also increased the risk of developing RA in women followed in the NHS I and II cohorts (HR = 1.35 (1.11–1.64) compared with normal BMI = 18.5–25 kg/m^2^). In these two cohorts, the mean age of diagnosis of RA was 57.9 years in NHS I and 47.6 years in NHS II [7]. In this same study, the duration of exposure to obesity seemed to play a role since 10 years of obesity was associated with a 37% increased risk of developing RA [7]. This corroborates our results, suggesting the impact of long-term fatty tissue excess early in life on the risk of subsequent RA.

In obesity, both immune cells and adipocytes infiltrate fat tissue. Adipocytes secrete high levels of some adipokines, responsible for a pro-inflammatory state [29]. As for example, leptin released by the fat tissue and thus increased in obese subjects, seems to be an important adipokine because of its metabolic and immunological properties. Leptin is a mediator of both innate and acquired immune responses by its pro-inflammatory effects: proliferation of monocytes and activation of macrophages phagocytosis, production of pro-inflammatory cytokines (such as TNFα, IL-6, etc.), stimulation of T and B cells proliferation, inhibition of T-regulatory cells proliferation, and mediation of immune tolerance [29,30]. Interestingly, in RA patients, levels of some pro-inflammatory adipokines such as leptin, resistin, and visfatin are increased in serum and synovial tissues [29,31].

Another mechanism to consider is the imbalance of the gut microbiota (dysbiosis) in case of obesity, probably at least partly due to the Western diet, rich in pro-inflammatory nutrients. Dysbiosis may alter the immunomodulatory role of the gut, with local and extra-intestinal effects on immune response [29]. Interestingly, Nguyen et al. found that chronic diarrhea (that may be due to dysbiosis) was associated with an increased risk of RA among women included in E3N cohort [32].

Among women, changes in body shape through ages were suggested to reflect pubertal growth and lifetime female hormonal changes from puberty to menopause, corresponding to the reproductive period [33]. Puberty is accompanied by a growth spurt, body composition changes, including the regional distribution of body fat. The hormonal regulation of the growth spurt and the changes in body composition depend on the release of gonadotropins, leptin, sex-steroids, and growth hormone. Moreover, estrogen levels and their receptors, as well as progestogen, play a role in fat distribution during the reproductive period, the weight gain, and the increase in visceral fat at menopause [33]. It is well known that obesity (especially abdominal obesity) reduces fertility and increases abortion risk in women due to “low-grade inflammatory state”, functional hyperandrogenism, and hyperinsulinemia and that these affect ovarian steroidogenesis, blood concentrations, and central regulations [34]. This inflammatory state, due to an excess of pro-inflammatory adipokines in obese subjects, may increase the risk of auto-immune diseases such as RA, especially in part due to a deregulation of sexual hormones in women [3,9]. 

Our study has some limitations. In the E3N cohort, measures of BS were self-reported, but the use of the eight Sørensen BS drawings was validated in a dedicated study [20]. In this study, the correlation between BMI and BS was 0.78. It was demonstrated that self-perception of the BS was “higher” among women who were overweight. Interestingly, women who had experienced such weight problems during adolescence had a more realistic appreciation of their current body image [20]. Our cohort included women aged 40 to 65 years at recruitment, and we considered only incident RA; thus, results cannot be extrapolated to RA occurring earlier in life. Moreover, our results on anthropometric measures might not be generalizable to men, where abdominal distribution of fatty excess is the rule. Additionally, we were unable to analyze data by auto-antibodies status, which was unknown for most of the cohort, but we did consider smoking status, which is highly associated with ACPA status. 

Strengths include the large cohort size, the rigorous and multi-source validation process for RA diagnoses, long follow-up period, and high response rates to questionnaires [14,15]. The rates of missing data were generally low and did not differ between cases and non-cases [15]. Moreover, with our prospective design, anthropometric measures as well as other covariates were collected rigorously and repeatedly before RA onset and thus were not subject to recall/memory bias. Moreover, some exposures could be analyzed as time-dependent variables. In addition, environmental factors, including those known to be associated with risk of RA, were considered in multivariable-adjusted models.

## 5. Conclusions

We report, for the first time, independent associations between RA and body shape throughout life. Medium body shape at puberty and medium-large body shape at perimenopause were associated with a moderately increased risk of RA. In addition, a constantly large body shape trajectory from puberty to perimenopause among women never exposed to smoking was associated with a 2-fold increased risk of incident RA. Our results suggest the role of fat tissue in the physiopathology of RA. They highlight that fat distribution rather than being overweight itself is associated with the risk of RA, especially when this “at-risk” fat distribution profile is maintained throughout life.

## Figures and Tables

**Table 1:** Hazard ratios (95% confidence intervals) for the risk of RA by BMI and abdominal obesity in multi-adjusted model (for the overall population, ever-smokers, and never-smokers).

Body Mass Index (BMI in kg/m^2^) *									
	**All Population (*N* = 78,452)**			**Ever-Smokers (*N* = 41,816)**			**Never-Smokers (*N* = 36,634)**		
	**RA**	**Non Cases**	**HRs (95% CI)**	**RA**	**Non Cases**	**HRs (95% CI)**	**RA**	**Non Cases**	**HRs (95% CI)**
BMI < 18.5	17	2874	0.75 (0.5–1.2)	10	1575	0.73 (0.4–1.4)	7	1299	0.80 (0.4–1.7)
BMI = 18.5–25	455	49,253	Ref	266	26,091	Ref	187	23,162	Ref
BMI = 25–30	171	19,690	1.10 (0.9–1.3)	97	10,448	1.10 (0.8–1.3)	74	9242	1.10 (0.8–1.4)
BMI ≥ 30	55	5937	1.26 (0.9–1.5)	35	3294	1.35 (0.9–1.9)	20	2643	1.15 (0.7–1.8)
*ptrend*			0.0559			0.0736			0.3487
Abdominal Obesity (Waist Circumference >88 cm) *^,†^									
	**All Population (*N* = 78,379)**			**Ever-Smokers (*N* = 41,771)**			**Never-Smokers (*N* = 36,608)**		
	**RA**	**Non Cases**	**HRs (95% CI)**	**RA**	**Non Cases**	**HRs (95% CI)**	**RA**	**Non Cases**	**HRs (95% CI)**
No	300	34,976	Ref	170	18,477	Ref	128	16,530	Ref
Yes	139	15,582	1.25 (1.0–1.5)	85	8527	1.32 (0.9–1.8)	54	7146	1.12 (0.8–1.6)
*p*-value			0.0338			0.0697			0.5367

HRs: hazard ratio; 95% CI: 95% confidence interval; Ref: reference. Ever-smokers: women exposed to tobacco during childhood and/or ever-smokers; never-smokers: women never exposed to tobacco during childhood and never-smokers. * time-dependent variables. ^†^ From 1994 questionnaire (Q4), updated at Q7, Q9, Q10, and Q11 and according to the WHO recommended cutoff values for women: waist circumference was >88 cm (including 627 RA). Waist circumference was missing for 27,384 (34.9%). Totals do not add up because missing values were deleted for age at the beginning of smoking among 2 women with RA (0.3%). Multi-adjusted model is stratified by year of birth and included age, smoking (past/current/never, except for women never exposed to smoking), passive smoking during childhood and/or adulthood (ever/never, except for women never exposed to smoking), educational level (<high school, up to 2 years of university, ≥3 years of university), baseline physical activity in MET.h/week (in quartiles), age at menarche (<13, 13–15, ≥15 years), age at menopause (≤45, 45–53, ≥53 years), number of full-term pregnancies (≤1, 2, ≥3), body mass index from Q1 to Q11 (<18.5, 18.5–25, 25–30, ≥30 kg/m^2^), and abdominal obesity from Q4 to Q11 (waist circumference >88 cm).

**Table 2:** Hazard ratios (95% confidence intervals) for the risk of RA by body shapes at puberty and at baseline in multi-adjusted model (for the overall population, ever-smokers, and never-smokers).

	All Population (*N* = 78,452)			Ever-Smokers (*N* = 41,816)			Never-Smokers (*N* = 36,634)		
	RA	Non Cases	HRs (95% CI)	RA	Non Cases	HRs (95% CI)	RA	Non Cases	HRs (95% CI)
Body shape at puberty									
Lean	332	40,485	Ref	183	20,423	Ref	148	20,062	Ref
Medium	179	17,903	1.22 (1.0–1.5)	103	9835	1.17 (0.9–1.5)	75	8068	1.30 (1.0–1.7)
Large	147	15,984	1.10 (0.90–1.4)	92	9511	1.10 (0.8–1.4)	55	6473	1.14 (0.8–1.6)
*ptrend*			0.1760			0.3899			0.2358
Body shape at baseline (perimenopausal period)									
Lean	359	44,718	Ref	220	23,804	Ref	138	20,914	Ref
Medium	200	20,041	1.15 (0.9–1.4)	103	10,489	0.94 (0.7–1.2)	96	9552	1.46 (1.1–1.9)
Large	110	9838	1.17 (0.8–1.5)	63	5330	1.0 (0.7–1.4)	47	4508	1.45 (0.9–2.2)
*ptrend*			0.1956			0.8268			0.0375

HRs: hazard ratios; 95% CI: 95% confidence interval; Ref: reference. Ever-smokers: women exposed to tobacco during childhood and/or ever-smokers; never-smokers: women never exposed to tobacco during childhood and never-smokers. Totals do not add up because missing values were deleted for age at the beginning of smoking among 2 women with RA (0.3%) and for body shape at puberty (*n* = 3422; 4.4%) and for body shape at baseline (*n* = 3186; 4.1%). Multi-adjusted model is stratified by year of birth and included age, smoking (past/current/never, except for women never exposed to smoking), passive smoking during childhood and/or adulthood (ever/never, except for women never exposed to smoking), educational level (<high school, up to 2 years of university, ≥3 years of university), baseline physical activity (in quartiles), age at menarche (<13, 13–15, ≥15 years), age at menopause (≤45, 45–53, ≥53 years), number of full-term pregnancies (≤1, 2, ≥3), baseline physical activity in MET.h/week (quartiles), both BS at puberty and BS at baseline, and body mass index from Q1 to Q11 (<18.5, 18.5–25, 25–30, ≥30 kg/m^2^).

**Table 3:** Hazard ratios (95% confidence intervals) for the risk of RA by body shape trajectories in multi-adjusted model (for the overall population, ever-smokers and never-smokers).

	All Population (*N* =77,552) ^α^			Ever-Smokers (*N* = 41,339)			Never-Smokers (*N* = 36,221)		
	RA	Non Cases	HRs (95% CI)	RA	Non Cases	HRs (95% CI)	RA	Non Cases	HRs (95% CI)
Body shape trajectories from puberty to perimenopausal period									
Constantly lean	101	12,415	Ref	59	6182	Ref	42	6233	Ref
Medium BS at puberty and sharp decrease	114	13,477	1.04 (0.8–1.4)	67	7314	0.97 (0.7–1.4)	46	6163	1.14 (0.7–1.7)
Large BS at puberty and decrease	152	19,780	1.0 (0.8–1.3)	98	10,868	1.0 (0.7–1.5)	54	8912	0.92 (0.6–1.4)
Constantly medium	119	12,023	1.10 (0.8–1.4)	57	6010	0.87 (0.6–1.3)	62	6013	1.44 (0.9–2.2)
Upper midrange	150	14,826	1.18 (0.9–1.5)	91	8103	1.07 (0.7–1.5)	58	6723	1.33 (0.9–2.0)
Constantly large	51	4344	1.29 (0.9–1.9)	26	2464	0.9 (0.5–1.5)	25	1880	2.10 (1.2–3.6)
*ptrend*			0.1243			0.9595			0.0248

HRs: hazard ratios; 95% CI: 95% confidence interval. Ref: reference. ^α^ Population included 77,552 women and 687 incident RA; 900 women were excluded because of missing data on all age-related BS. Ever-smokers: women exposed to tobacco during childhood and/or ever-smokers; never-smokers: women never exposed to tobacco during childhood and never-smokers. Two women were excluded from the stratified analyses because age at the beginning of smoking was missing. Multi-adjusted model is stratified by year of birth and included age, smoking (past/current/never, except for women never exposed to smoking), passive smoking during childhood and/or adulthood (ever/never, except for women never exposed to smoking), educational level (<high school, up to 2 years of university, ≥3 years of university), baseline physical activity (in quartiles), age at menarche (<13, 13–15, ≥15 years), age at menopause (≤45, 45–53, ≥53 years), number of full-term pregnancies (≤1, 2, ≥3), baseline physical activity in MET.h/week (quartiles), body mass index from Q1 to Q11 (<18.5, 18.5–25, 25–30, ≥30 kg/m^2^), and body shape trajectories.

## Data Availability

Data are available upon reasonable request.

## Supplementary Materials

The following supporting information can be downloaded at: https://www.mdpi.com/article/10.3390/nu14050934/s1, Table S1: Baseline characteristics of study population; Table S2: Cox proportional-hazards analysis for RA by anthropometric features.
