# Supplementary material for: Anthropometric Measures and Risk of Rheumatoid Arthritis in the French E3N Cohort Study
Source: Nutrients. 2022 Feb 22;14(5):934. doi: 10.3390/nu14050934 (PMC8912452; doi:10.3390/nu14050934)
Supplement: Supplementary file 1 [file nutrients-14-00934-s001.zip › nutrients-1577882-supplementary.pdf]

Table S1: Baseline characteristics of study population

		All (N=78,452)	Incident RA (N=698)
Age at baseline (in years)	Mean (±SD)	49.0 (±6.4)	49.0 (±6.2)
Year of birth	Before 1930	4,877 (6.2)	39 (55.9)
	1930-1935	9,421 (12.0)	14 (2)
	1935-1940	15,025 (19.1)	146 (21)
	1940-1945	19,600 (25.0)	191 (27.4)
	After 1945	29,528 (37.7)	208 (29.8)
Smoking status*	Current	11,120 (14.2)	120 (17.2)
	Never	42,394 (54.0)	350 (50.1)
	Past	24,938 (31.8)	228 (32.7)
Passive smoker during childhood and/or adulthood			
	No	32,216 (41.1)	256 (36.7)
	Yes	46,236 (58.9)	442 (63.3)
Physical activity (MET.h / week)	Mean (SD)	42.9 (±20.9)	43.2 (±21.1)
Educational level	< High School	11,693 (15.0)	125 (17.9)
	Up to 2 level university	39,639 (50.5)	351 (50.3)
	≥ 3 level university	27,120 (34.6)	222 (31.8)
**REPRODUCTIVE FACTORS**			
Age at menarche	Mean (±SD)	12.8 (±1.4)	12.7 (±1.4)
Number of full-term pregnancies	Mean (±SD)	2.0 (±1.1)	1.9 (±1.1)
Menopausal status at baseline	No	48,092 (61.3)	393 (1)
	Yes	30,360 (38.7)	305 (1)
Age at menopause among menopausal women at baseline (in years) (N=30,360)			
	Mean (±SD)	48.9 (±4.6)	48.2 (±5.0)
**ANTHROPOMETRIC MEASURES**		**All (N=78,452)**	**Incident RA (N=698)**
Body shape at 8 years	Lean	40,023 (51.0)	364 (52.1)
	Medium	15,578 (19.8)	126 (18.0)
	Large	18,234 (23.2)	156 (22.3)
	Missing	4,617 (5.9)	52 (7.4)
Body shape at puberty	Lean	40,817 (52.0)	332 (47.5)
	Medium	18,082 (23.0)	179 (25.6)
	Large	16,131 (20.6)	147 (21.0)
	Missing	3,422 (4.4)	40 (5.7)
Body shape at 20-25 years	Lean	39,663 (50.5)	345 (49.4)
	Medium	25,615 (32.6)	215 (30.8)
	Large	10,650 (13.6)	108 (15.5)

	Missing	2,524 (3.2)	30 (4.3)
**ANTHROPOMETRIC MEASURES**		**All (N=78,452)**	**Incident RA (N=698)**
Body shape at 35-40 years	Lean	24,570 (31.3)	184 (26.4)
	Medium	32,289 (41.2)	266 (38.1)
	Large	18,958 (24.2)	215 (30.8)
	Missing	2,635 (3.3)	33 (4.7)
Body Shape at baseline	Lean	45,077 (57.5)	359 (51.4)
	Medium	20,241 (25.8)	200 (28.6)
	Large	9,948 (12.7)	110 (15.7)
	Missing	3,186 (4.1)	29 (4.1)
Trajectories of body shape over the life course (from puberty to perimenopause)[α]			
	Constantly lean BS	12,516 (16.1)	101 (14.7)
	Medium BS at puberty/sharp decrease in BS after puberty	13,591 (17.5)	114 (16.6)
	Large BS at puberty/decrease in BS after puberty	19,932 (25.7)	152 (22.1)
	Constantly medium BS	12,142 (15.7)	119 (17.3)
	Upper midrange BS	14,976 (19.3)	150 (21.8)
	Constantly large BS	4,395 (5.7)	51 (7.4)
Baseline Body mass index (kg/m²)*	< 18.5	3,280 (4.2)	27 (3.8)
	[18.5-25[	62,693 (79.9)	540 (77.3)
	[25-30[	10,455 (13.3)	111 (15.9)
	≥ 30	2,024 (2.6)	24 (3.4)
Waist circumference (cm)[δ]	<70	13,048 (16.6)	108 (16.9)
	[70-75]	19,317 (24.6)	130 (20.4)
	]75-80]	11,996 (15.3)	90 (14.1)
	>80	14,028 (17.9)	123 (19.3)
	Missing	19,992 (25.5)	176 (27.6)
Hip circumference (cm)[δ]	<91	13,049 (16.6)	128 (20.1)
	[91-96]	17,942 (22.9)	98 (15.4)
	]96-101]	13,116 (16.7)	98 (15.4)
	>101	14,205 (18.1)	125 (19.6)
	Missing	20,070 (25.6)	178 (27.9)
Abdominal obesity according to waist circumference†			
	No	53,330 (68.0)	405 (63.6)
	Yes	5,059 (6.4)	46 (7.2)
	Missing	19,992 (25.5)	176 (27.6)
Abdominal obesity according to the waist-to-hip circumference ratio[β]			
	No	51,592 (65.8)	395 (62.0)

	Yes	6,606 (8.4)	53 (8.3)
	Missing	20,183 (25.7)	179 (28.1)

SD: standard deviation, BS: body shape, MET.h / week: metabolic equivalents of task, hours per week.
$^{\alpha}$ Population included 77,552 women and 687 incident RA, 900 women were excludes because of missing data on all age-related BS.
*Time dependent variables from Q1 to Q11.
$^{\delta}$ From 1994 questionnaire (Q4) included 78,381 women with 627 RA, in quartiles.
†From 1994 questionnaire (Q4) and according to World Health Organization (WHO) recommended cutoff value: waist circumference>88 cm for women.
$^{\beta}$From 1994 questionnaire (Q4), according to the WHO cutoff value for abdominal obesity: waist-to-hip ratio>0.85 for women.

TABLE S2: Cox proportional-hazards analysis for RA by anthropometric features

	RA (N=698)	Non-cases (N=77,754)	Hazard Ratios (95% Confidence Interval) MODEL 1	*ptrend*	MODEL 2	*ptrend*
**Body mass index from Q1 to Q11 (kg/m²)**						
<18.5	17	2,874	0.76 (0.5-1.4)		0.76 (0.4-1.4)	
[18.5-25[	455	49,253	Ref	***0.0263***	Ref	***0.0445***
[25-30[	171	19,690	1.10 (0.9-1.3)		1.10 (0.9-1.3)	
**≥ 30**	55	5,937	**1.33 (1.0-1.8)**		**1.30 (1.0-1.7)**	
**Abdominal obesity according to waist circumference†**						
≤88 cm	300	34,976	**Ref**		**Ref**	
**>88 cm**	139	15,582	**1.30 (1.0-1.6)**		**1.23 (1.0-1.5)**	
Abdominal obesity according to waist-to-hip ratio†						
≤0.85	285	29,942	Ref		Ref	
>0.85	153	19,807	1.04 (0.8-1.3)		1.0 (0.8-1.2)	
Body shape at 8 years						
Lean	364	39,659	ref		Ref	
Medium	126	15,452	0.91 (0.7-1.1)	*0.5819*	0.91 (0.7-1.1)	*0.4832*
Large	156	18,078	0.96 (0.8-1.1)		0.95 (0.8-1.1)	
**Body shape at puberty**						
Lean	332	40,485	Ref		Ref	
**Medium**	179	17,903	**1.23 (1.0-1.5)**	*0.0794*	**1.22 (1.0-1.5)**	*0.1361*
Large	147	15,984	1.15 (0.9-1.4)		1.12 (0.9-1.4)	
Body shape at 20-25 years						
Lean	345	39,318	Ref		Ref	
Medium	215	25,400	0.98 (0.8-1.2)	*0.2518*	0.97 (0.8-1.1)	*0.4369*
Large	108	10,542	1.18 (0.9-1.5)		1.14 (0.9-1.5)	
Body shape at 35-40 years						
Lean	215	24,355	1.04 (0.9-1.2)		1.07 (0.9-1.3)	
Medium	266	32,023	Ref	*0.2466*	Ref	*0.7293*
Large	184	18,774	1.18 (1.0 -1.4)		1.13 (0.9-1.4)	
**Body shape at baseline**						
Lean	359	44,718	Ref		Ref	
**Medium**	200	20,041	**1.20 (1.0-1.4)**	***0.0055***	1.17 (0.9-1.4)	*0.0637*
**Large**	110	9,838	**1.32 (1.1-1.6)**		1.26 (0.9-1.6)	

	RA (N=687)	Non-cases (N=76,865)	Hazard Ratios (95% Confidence Interval) MODEL 1	MODEL 2
**Trajectories of body shape over the life course (puberty to perimenopause)**				
Constantly lean BS	101	12,415	Ref	Ref
Medium BS at puberty/sharp decrease in BS after puberty	114	13,477	1.04 (0.8-1.4)	1.05 (0.8-1.4)
Large BS at puberty/decrease in BS after puberty	152	19,780	1.01 (0.8-1.3)	1.0 (0.8-1.3)
Constantly medium BS	119	12,023	1.15 (0.9-1.5)	1.11 (0.8-1.5)
Upper midrange BS	150	14,826	1.24 (0.9-1.6)	1.20 (0.9-1.6)
**Constantly large BS**	51	4,344	**1.42 (1.0-2.0)**	1.31 (0.9-1.9)
ptrend			***0.0119***	*0.1027*

ptrend: p for trend. Ref: reference class.
†From 1994 questionnaire (Q4) and according to World Health Organization (WHO) recommended cutoff values for women: waist circumference was >88 cm or waist-to-hip ratio >0.85.
All models were stratified by year of birth.
BMI, smoking status and abdominal obesity were analyzed as time-dependent variables..
Model 1 is age-adjusted.
Model 2 is adjusted for age, smoking (past/current/never), passive smoking during childhood and/or adulthood (ever/never), educational level (<high–school, up to 2 years of university, ≥ 3 years of university), body mass index from Q1 to Q11 (<18.5, [18.5–25[, [25–30[, ≥30 kg/m²), excepted for BMI.
